# Topical metronidazole after haemorrhoidectomy to reduce postoperative pain: a systematic review

**DOI:** 10.1007/s13304-024-01930-3

**Published:** 2024-08-08

**Authors:** Chiara Eberspacher, Domenico Mascagni, Stefano Pontone, Francesco Leone Arcieri, Stefano Arcieri

**Affiliations:** Department of Surgery, University of Rome “Sapienza”, Viale Regina Elena 324, 00161 Rome, Italy

**Keywords:** Haemorrhoidectomy, Postoperative pain, Topical metronidazole, Systematic review

## Abstract

Excisional haemorrhoidectomy is the gold standard for operating haemorrhoids, but it is accompanied by a significant problem: postoperative pain. Several strategies have been adopted to minimize this condition. Oral metronidazole has been proven to reduce postoperative pain but with some complications. This systematic review was conducted to investigate the effects and general efficacy of topical metronidazole administration and to evaluate its potential superiority over the oral formula. A systematic review of the literature was carried out. Randomized controlled trials published until September 2023 on PubMed, Central, and Web of Science were considered. The primary outcome considered was postoperative pain, which was evaluated using visual analogue scores. The secondary outcomes were analgesic use, return to work, and complications. Six randomized controlled trials were included, with a total of 536 patients. Topical metronidazole was compared with placebo in two studies, with oral formula in three studies, and with placebo and oral administration in one study. Topical metronidazole was found to be effective for treating postoperative pain when compared to a placebo but had no significant advantage over the oral formula. No complications were reported in the studies. Topical and oral metronidazole are effective solutions for postoperative pain after excisional haemorrhoidectomy. No superiority was demonstrated based on the route of administration, and complications were marginal for both formulas. Further studies are required to determine the best metronidazole solution.

## Introduction

Haemorrhoidal disease is a common proctologic disease in the adult population, with an estimated incidence that widely varies from 4.4 to 39% based on the source of information [1–3]. The actual prevalence would be higher because many people do not require any therapy apart from conservative methods (diet, lifestyle modifications, stool softeners, etc.) and they do not report their symptoms at hospital or other healthcare centres. In the early stages of the disease, medical management is preferred [4]. Surgical operation is required when the nonoperative approach fails for grade III/IV of the disease, according to Goligher’s classification, or in the case of complications. Haemorrhoidectomy is considered the gold standard in surgery, especially with regard to the possibility of symptomatic recurrence [1, 5]. The main disadvantage is the presence of an extended wound in the sensitive part of the anus, with significant postoperative (PO) pain and a delay in the patient’s return to normal daily activities [1]. Various strategies to minimize PO pain have been studied, including the use of different excision techniques and tools (e.g. radiofrequency and ultrasonic devices) [6, 7] and the use of ointments (e.g. glyceryl trinitrate, local anaesthetics, steroids and sucralfate) postoperatively, but these strategies demonstrated variable outcomes [8]. In the 1990s, metronidazole was first used in oral form. The postulated principles were to decrease secondary bacterial colonization or infection and mediate the direct anti-inflammatory response [9, 10]. The transition to a topical ointment was guided by various purposes, such as maximizing local tissue concentrations and bioavailability and preventing systemic side effects [11].

This systematic review was conducted to summarize the use, efficacy, and possible complications of topical metronidazole ointment after haemorrhoidectomy based on previous clinical trials. The objective was to understand whether topical metronidazole is effective in diminishing PO pain after haemorrhoidectomy and if it is superior to oral formula in terms of pain control and side effects. This review was conducted according to the Preferred Reporting Items for Systematic Reviews and Meta-Analyses (PRISMA) guidelines [12].

## Methods

**Fig. 1:**
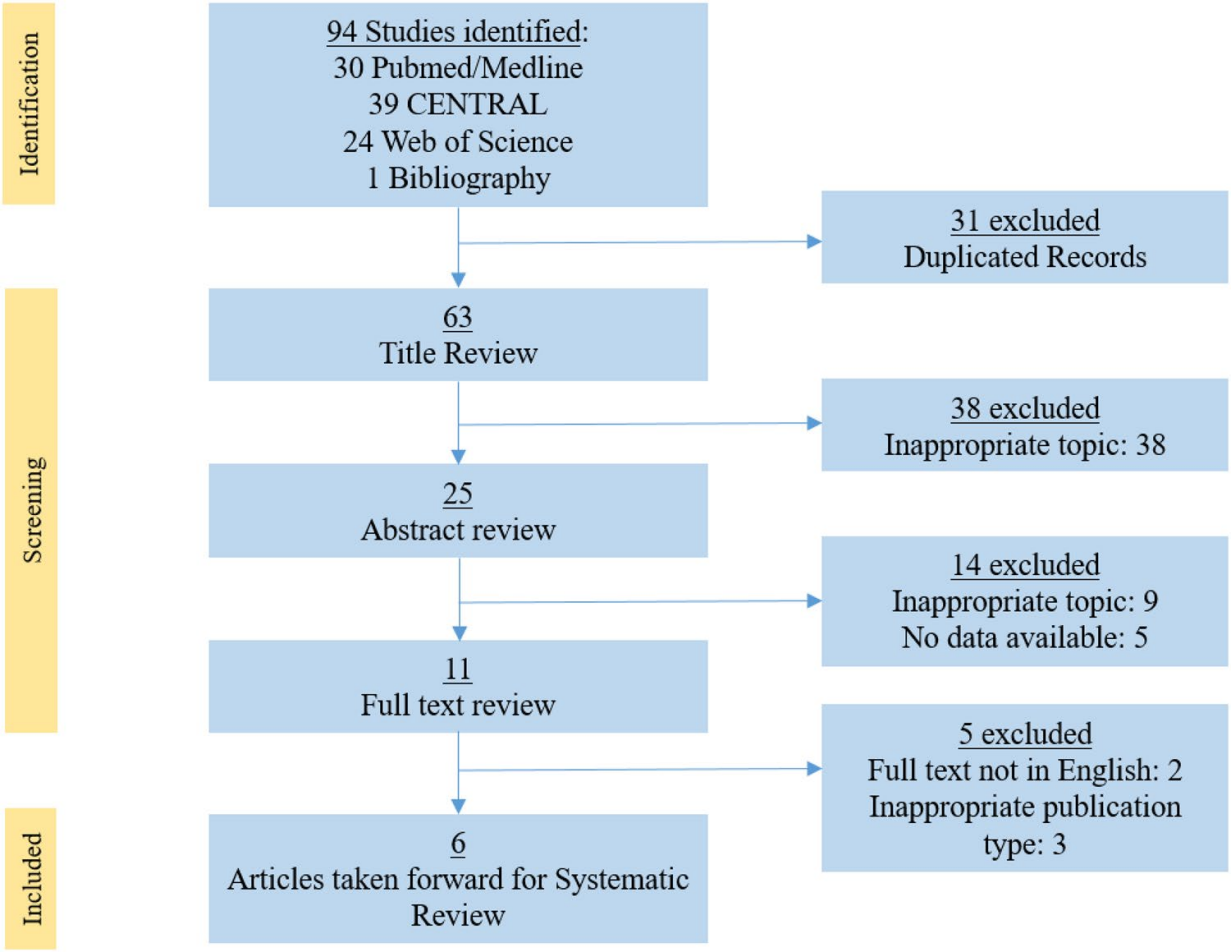
PRISMA flow chart

This systematic review was conducted in accordance with the PRISMA 2020 statement. Two authors independently created an electronic database for full text articles published in English and indexed in PubMed/MEDLINE, Cochrane Central Register of Controlled Trials (CENTRAL) and Web of Science Core Collection. Only randomised controlled trials (RCTs) and meta-analyses of RCTs investigating the use of metronidazole following haemorrhoidectomy were included. Further, only excision haemorrhoidectomy methods were considered, specifically the Milligan–Morgan and Ferguson techniques. The following search terms were used: (‘haemorrhoidectomy’ (title) OR ‘haemorrhoidectomy’ (title)) AND (‘postoperative pain’ (all fields) OR ‘post haemorrhoidectomy pain’ (title/abstract) OR ‘post haemorrhoidectomy pain’ (title/abstract)) AND (‘metronidazole’ (title/abstract) OR ‘Flagyl®’ (title/abstract)). Synonyms for each of these terms were also used in the search, which was limited to human trials. A manual review of the references of suitable studies was conducted to identify additional studies that the search strategy mentioned above might have missed. All studies comparing topical metronidazole with systemically administered metronidazole, with a placebo or with other ointments were included in this review. The exclusion criteria were the absence of an abstract, studies that were not RCTs, systematic reviews, or meta-analyses of RCTs, articles not in English, and studies without extractable data. Two independent authors (SP and FLA) selected studies that met the inclusion criteria. In case of disagreement, the principal investigator (CE) resolved the differences between the two authors. The last search was run on 15 September 2023. A total of 94 eligible articles were found (31 from PubMed/Medline, 39 from CENTRAL, and 24 from the Web of Science Core Collection). Finally, six RCTs were included in the present study. A PRISMA flow chart with the selection of the articles is shown in Fig. 1.

### Outcome of interest

The outcome of interest was post-haemorrhoidectomy pain, which was evaluated using the visual analogue scale (VAS). The other outcomes were analgesic consumption and metronidazole-related complications. As mentioned above, two authors created a database of the extracted data, including baseline characteristics of patients, number of patients, type of operation, type of anaesthesia used, type of intervention, PO analgesic therapy used and primary and secondary outcomes.

## Results

This systematic review included six RCTs involving 536 participants: 253 subjects randomly assigned to the topical metronidazole group (TMG) and 283 subjects comprising the control group (consisting of the placebo ointment group (PCG) and oral metronidazole-treated group (OMG)). Table 1 summarises the characteristics of the six RCTs.

**Table 1:** Baseline characteristics of the RCTs evaluating the use of topical metronidazole after haemorrhoidectomy

Study	Procedure	Anaesthesia performed	Haemorrhoid grade	Type of intervention	Total no. of patients (no. of patients in the topical metronidazole group)	Analgesic therapy used
Nicholson, 2004	Closed haemorrhoidectomy with harmonic scalpel	General anaesthesia	III–IV	Topical (10%) vs placebo	20 (10)	Hydrocodone (10 mg by mouth every 5–6 h as needed)
Ala, 2007	Haemorrhoidectomy	–	II, III, IV	Topical (10%) vs placebo	47 (25)	–
Neogi, 2018	Open haemorrhoidectomy	–	II, III, IV	Topical vs placebo vs oral	60 (20)	Analgesic tablets (?)
Razzaq, 2020	Milligan–Morgan	General anaesthesia	III–IV	Topical vs oral	120 (60)	–
Abbas, 2020	Milligan–Morgan	–	III–IV	Tropical vs oral	166 (83)	–
Xia, 2022	Milligan–Morgan/Ferguson with or without LigaSure or MarClamp	General anaesthesia + Pudendal nerve block	III–IV	Topical vs oral	92 (46)	Paracetamol (1 g 4 times/day) Ibuprofen (400 mg 3 times/day) Oral tramadol (for breakthrough pain)

In four studies (Table 2), topical metronidazole was superior to the placebo in addressing PO pain (primary outcome). Nicholson reported a significantly different mean VAS score for TMG and PCG (3.04 vs 6.03; *p* = 0.003), on PO day 7 [11]. This result was confirmed by Ala et al. and Neogi [13, 14]. Neogi reported a significantly lower mean VAS score in TMG than in PCG (1.55 vs 2.30; *p* = 0.0001) [14]. Razzaq also reported a superior pain control in TMG [15]. However, no evaluation was made on PO day 7; nevertheless, on PO days 1, 3 and 5, the TMG and PCG had significantly different VAS scores for the last two lapses of time (3.96 vs 2.74 with *p* = 0.024 and 3.08 vs 2.14 with *p* = 0.01).

**Table 2:** Results of the RCTs evaluating the use of topical metronidazole for post-haemorrhoidectomy pain

Study	PO day	VAS scores of the topical metronidazole group (mean ± SD)	VAS scores of the oral metronidazole group (mean ± SD)	VAS scores of the placebo group (mean ± SD)	*p*
Nicholson, 2004	1				> 0.05
	7	3.4 ± 0.4	–	6.3 ± 0.05	0.002
	14	1 ± 0.4	–	3.2 ± 0.7	0.02
	28				> 0.05
Ala, 2007	1	Qualitative data only	–	Qualitative data only	0.04
	2		–		< 0.01
	7		–		0.03
	14		–		< 0.01
Neogi, 2018					(ANOVA test)
	1	6.20 ± 0.76	4.30 ± 0.65	4.90 ± 0.71	0.006
	3	2.80 ± 0.76	3.15 ± 0.81	3.85 ± 0.74	0.000
	7	1.55 ± 0.51	1.55 ± 0.68	2.30 ± 0.57	0.0001
Razzaq, 2020	1	6.89 ± 1.76	–	6.42 ± 1.38	> 0.05
	3	3.96 ± 1.28	–	2.74 ± 1.06	0.024
	5	3.08 ± 0.24	–	2.14 ± 0.26	0.01
Abbas, 2020	7	3.04 ± 1.41	4.05 ± 1.92	–	0.01
Xia, 2022	3 (Generalized mixed linear model 0–7)	6.31 ± 0.39	5.84 ± 0.73	–	0.38

Three studies compared TMG with OMG. These groups showed no significant difference in pain control based on the findings of Neogi (who compared TMG, ORM and PCG) and Xia. Neogi and Xia reported that on PO day 3, the mean VAS scores for TMG vs OMG were 2.08 vs 3.15 and 6.31 vs 5.84, respectively, both with *p* > 0.05 [14, 16]. Only Abbas found that pain control was superior in TMG than that in OMG on PO day 7 (3.04 vs 4.05, *p* = 0.01) [17].

Comparing all the studies together is nearly impossible given that PO pain was evaluated on different days, and in some cases, VAS scores were not reported (Ala used only graphics).

Only four studies evaluated analgesic consumption as secondary outcome. Nicholson and Xia did not report any significant difference between the groups, whereas Ala and Neogi reported less consumption of topical analgesics in TMG [11, 13, 14, 16]. However, obtaining a uniform set of data for analgesic consumption is impossible (Table 3).

**Table 3:** Secondary outcomes evaluated in the RCTs

Study	Analgesic use				Complications				Other outcomes evaluated
	Topical	Placebo	Oral	*p*	Topical	Placebo	Oral	*p*	
Nicholson, 2004	Values not reported	Values not reported	–	0.32	No complications	No complications	–	–	Wound healing
Ala, 2007	Values not reported	Values not reported	–	*p* < 0.05	6	5	–	*p* > 0.05	–
Neogi, 2018	2.2 ± 2.01	8.95 ± 5.11	3.85 ± 3.57	*p* < 0.05	–	–	–	–	–
Razzaq, 2020	–	–	–	–	–	–	–	–	–
Abbas, 2020	–	–	–	–	–	–	–	–	–
Xia, 2022	Values not reported	–	Values not reported	*p* > 0.05	7	–	9	*p* = 0.79	Bowel function Recovery Score Preference and compliance

Complications related to treatment with metronidazole in any formulation, which is another secondary outcome, were reported only by Ala and Xia, who showed no explicit correlation between the complications observed and metronidazole treatment [13, 16].

Only a few studies have assessed other secondary outcomes, namely, pain in the healing wound, pain when defecating, bowel function, recovery score and compliance (Table 3).

## Discussion

PO pain after haemorrhoidectomy remains a challenging problem. The strategies adopted to minimize this problem include different methods of anaesthesia, a variety of surgical techniques, intraoperative complements, and postoperative solutions [18]. The use of oral metronidazole to decrease PO pain after haemorrhoidectomy is not a recent finding. Conducting an RCT in 1998, Carpeti et al. demonstrated that pain control after a diathermic haemorrhoidectomy may be improved using oral metronidazole [10]. The mechanism that led to this result is still only a hypothesis: bacterial colonization decreased in the operation site, especially after open haemorrhoidectomy or Milligan-Morgan. The postulated occurrence of significant bacterial colonization of wounds after haemorrhoidectomy is still under debate, including the nature of this colonization. Paula et al. identified Escherichia coli without any obliged anaerobia in "not infected" PO wounds, whereas other researchers have found anaerobic bacteria in infected wounds [19, 20]. The presence of an anomalously large number of bacteria may delay wound healing and, in some rare and episodic cases, may cause general sepsis [21].

Due to its effective action against anaerobes, metronidazole is undoubtedly preferred as treatment in anorectal conditions, such as perianal Crohn's disease in the paediatric population [22]. Furthermore, metronidazole exerts an anti-inflammatory action in some dermatological conditions. A study demonstrated that in patients with rosacea, metronidazole decreases the levels of reacting oxygen species and indirectly modulates neutrophil action, providing protection against auto-oxidative tissue damage [23].

Although the mechanism of oral metronidazole in decreasing pain after haemorrhoidectomy is not yet clear, randomized studies and meta-analyses have evidenced its efficacy in decreasing pain [24, 25]. The uncertainty surrounding the use of this antibiotic, even at low dosages, revolves around its collateral effects, such as nausea, anorexia and taste alteration [26]. Another argument against its use is its potential negative impact on gut microbiota [27, 28]. The formulation of topical metronidazole has been considered a strategy to avoid these complications as well as to achieve other benefits, such as increased local bioavailability and patient compliance.

Nicholson et al. deserve credit for being the first to analyse the efficacy of topical metronidazole in 2004, with the study group exhibiting better results than the placebo group [11]. A significant difference in pain was first observed on PO day 7, suggesting that the pain experienced on PO day 1 was due to the surgical operation rather than due to inflammatory response. However, the effect of metronidazole during the first week was relevant given the contribution of bacterial colonisation to the pain mechanism. Another significant contribution of their study was the evaluation of wound healing, along with the modality used for evaluation: three blinded observers evaluated healing and oedema from the digital pictures of PO wounds acquired during follow-up. This is the first and only study that considers the possibility that metronidazole, given its ability to modulate local oedema, affects the evolution of incisional margins in skin tags. Despite the innovative idea behind this study, its validity was limited by the small sample size; only 10 patients were assessed in each group, and no significant difference was found in their consumption of analgesics. The subsequent studies partly confirmed the efficacy of topical metronidazole in reducing PO pain. Specifically, the efficacy observed in TMG was superior to that observed in the control group in Ala’s and Neogi’s studies, which involved large sample sizes [13, 14]. The problems in these studies were the extreme variability in which VAS scores were determined during the PO period and the lack of uniformity. Xia conducted the only rigorous RCT, and no significant differences were observed between the topical and oral formulations in terms of the primary and secondary outcomes [16].

Another problem in evaluating the efficacy of topical metronidazole is that only a few studies have reported on secondary outcomes, such as analgesic consumption, and those studies are quite variable in terms of the type of analgesic used and route of administration, among others.

The limitation of this review is that it is impossible to achieve data uniformity that would establish a significant consideration on the use of topical metronidazole. The RCTs demonstrated that a topical formulation can indeed reduce pain compared with a placebo, but its comparison with an oral formulation remains incomplete.

## Conclusion

This systematic review showed that topical metronidazole effectively reduces post-haemorrhoidectomy pain compared with a placebo. Only one study demonstrated that the topical formulation is more effective for PO pain control than the oral formulation. The ‘expected’ increase in the bioavailability and efficacy of a topical formulation is thus an object of study for future research. This review evaluated only six RCTs on topical metronidazole, some of which have essential limitations, such as uniformity in the sex of patients and small sample size. Additional RCTs are needed to verify the equivalent pain control efficacies of topical and oral metronidazole formulations and their superiority over other ointments and strategies.

## Data Availability

The data that support the findings of this study are available on request from the corresponding author.

## Abbreviations

PO: Postoperative pain
VAS: Visual analogue score
PRISMA: Preferred Reporting Items for Systematic Reviews and Meta-Analyses
RCT: Randomised controlled trial
TMG: Topical metronidazole group
PCG: Placebo control group
OMG: Oral metronidazole group

## References

[1] Gallo G, Martellucci J, Sturiale A, Clerico G, Milito G, Marino F, Cocorullo G, Giordano P, Mistrangelo M, Trompetto M (2020) Consensus statement of the Italian society of colorectal surgery (SICCR): management and treatment of hemorrhoidal disease. Tech Coloproctol 24(2):145–164. 10.1007/s10151-020-02149-1 31993837 10.1007/s10151-020-02149-1 PMC7005095

[2] Lohsiriwat V (2012) Hemorrhoids: from basic pathophysiology to clinical management. World J Gastroenterol 18(17):2009–2017. 10.3748/wjg.v18.i17.2009 22563187 10.3748/wjg.v18.i17.2009 PMC3342598

[3] Peery AF, Sandler RS, Galanko JA, Bresalier RS, Figueiredo JC, Ahnen DJ, Barry EL, Baron JA (2015) Risk factors for hemorrhoids on screening colonoscopy. PLoS ONE 10(9):e0139100. 10.1371/journal.pone.0139100 26406337 10.1371/journal.pone.0139100 PMC4583402

[4] Stratta E, Gallo G, Trompetto M (2021) Conservative treatment of hemorrhoidal disease. Rev Recent Clin Trials 16(1):87–90. 10.2174/15748871156662010211501445 33087033 10.2174/15748871156662010211501445

[5] Lumb KJ, Colquhoun PH, Malthaner RA, Jayaraman S (2006) Stapled versus conventional surgery for hemorrhoids. Cochrane Database Syst Rev. 2006(4):CD005393. 10.1002/14651858.CD005393.pub2. (**PMID: 17054255; PMCID: PMC8887551**) 17054255 10.1002/14651858.CD005393.pub2 PMC8887551

[6] Milito G, Lisi G, Aronadio E, Campanelli M, Venditti D, Grande S, Grande M (2017) LigasureTM hemorrhoidectomy: how we do. Minerva Gastroenterol Dietol 63(1):44–49. 10.23736/S1121-421X.16.02343-6 27768009 10.23736/S1121-421X.16.02343-6

[7] Eberspacher C, Mascagni P, Di Nardo D, Pironi D, Pontone S, Martellucci J, Naldini G, Mascagni D (2020) Caiman Versus LigaSure hemorrhoidectomy: postoperative pain, early complications, long-term follow-up, and costs. Surg Innov 27(3):272–278. 10.1177/1553350620908388. (**Epub 2020 Mar 5 PMID: 32133936**) 32133936 10.1177/1553350620908388

[8] Cheetham MJ, Phillips RK (2001) Evidence-based practice in haemorrhoidectomy. Colorec Dis 3(2):126–134. 10.1046/j.1463-1318.2001.00189.x 10.1046/j.1463-1318.2001.00189.x 12791006

[9] Freeman CD, Klutman NE, Lamp KC (1997) Metronidazole. A therapeutic review and update. Drugs 54(5):679–708. 10.2165/00003495-199754050-00003 9360057 10.2165/00003495-199754050-00003

[10] Carapeti EA, Kamm MA, McDonald PJ, Phillips RK (1998) Double-blind randomised controlled trial of effect of metronidazole on pain after day-case haemorrhoidectomy. Lancet 351(9097):169–172. 10.1016/S0140-6736(97)09003-X 9449871 10.1016/S0140-6736(97)09003-X

[11] Nicholson TJ, Armstrong D (2004) Topical metronidazole (10 percent) decreases posthemorrhoidectomy pain and improves healing. Dis Colon Rectum 47(5):711–716. 10.1007/s10350-003-0129-z 15054681 10.1007/s10350-003-0129-z

[12] Page MJ, McKenzie JE, Bossuyt PM, Boutron I, Hoffmann TC, Mulrow CD, Shamseer L, Tetzlaff JM, Akl EA, Brennan SE, Chou R, Glanville J, Grimshaw JM, Hróbjartsson A, Lalu MM, Li T, Loder EW, Mayo-Wilson E, McDonald S, McGuinness LA, Moher D (2021) The PRISMA 2020 statement: an updated guideline for reporting systematic reviews. BMJ 372:71. 10.1136/bmj.n71 10.1136/bmj.n71 PMC8005924 33782057

[13] Ala S, Saeedi M, Eshghi F, Mirzabeygi P (2008) Topical metronidazole can reduce pain after surgery and pain on defecation in postoperative hemorrhoidectomy. Dis Colon Rectum 51(2):235–238. 10.1007/s10350-007-9174-3 18176825 10.1007/s10350-007-9174-3

[14] Neogi P, Sinha A, Singh M (2018) (2018) Is metronidazole a panacea for post-hemorrhoidectomy pain? Int Surg J 5:3598–3601 10.18203/2349-2902.isj20184629

[15] Razzaq S, Khan Z, Mahmood MA, Khan MN, Iqbal W, Zareen N (2021) Comparison of effectiveness of topical and oral metronidazole for reducing postoperative pain after hemorrhoidectomy. Med Forum Monthly. 2020, 31(10), 111‐113 | added to CENTRAL: 31 May 2021 | 2021 Issue 05

[16] Xia W, Barazanchi AWH, MacFater WS, MacCormick AD, Svirskis D, Sammour T, Hill AG (2022) Topical versus oral metronidazole after excisional hemorrhoidectomy: a double-blind randomized controlled trial. Dis Colon Rectum 65(11):1362–1372. 10.1097/DCR.0000000000002163 34897211 10.1097/DCR.0000000000002163

[17] Abbas ST, Raza A, MuhammadCh I, Hameed T, Hasham N, Arshad N (2020) Comparison of mean pain score using topical and oral metronidazole in post Milligan Morgan hemorrhoidectomy patient; a randomized controlled trial. Pakistan J Med Sci 36(5):867–871. 10.12669/pjms.36.5.1796 10.12669/pjms.36.5.1796 PMC7372682 32704254

[18] Lohsiriwat V, Jitmungngan R (2022) Strategies to reduce post-hemorrhoidectomy pain: a systematic review. Medicina 58(3):418. 10.3390/medicina58030418 35334594 10.3390/medicina58030418 PMC8955987

[19] de Paula PR, Speranzini MB, Hamzagic HC, Bassi DG, Chacon-Silva MA, Novo NF, Goldenberg S (1991) Bacteriology of the anal wound after open hemorrhoidectomy. Qualitative and quantitative analysis. Dis Colon Rectum 34(8):664–669. 10.1007/BF02050347 1855423 10.1007/BF02050347

[20] Brook I, Frazier EH (1996) Aerobic and anaerobic microbiology of infected hemorrhoids. Am J Gastroenterol 91(2):333–335 8607502

[21] Lee CY, Lee YJ, Chen CC, Kuo LJ (2021) Streptococcal toxic shock syndrome after hemorrhoidectomy: a case report. World J Clin Cases 9(33):10238–10243. 10.12998/wjcc.v9.i33.10238 34904094 10.12998/wjcc.v9.i33.10238 PMC8638029

[22] Zalieckas JM (2017) Treatment of perianal Crohn’s disease. Semin Pediatr Surg 26(6):391–397. 10.1053/j.sempedsurg.2017.10.009 29126509 10.1053/j.sempedsurg.2017.10.009

[23] Miyachi Y, Imamura S, Niwa Y (1986) Anti-oxidant action of metronidazole: a possible mechanism of action in rosacea. Br J Dermatol 114(2):231–234. 10.1111/j.1365-2133.1986.tb02802.x 2936372 10.1111/j.1365-2133.1986.tb02802.x

[24] Lyons NJR, Cornille JB, Pathak S, Charters P, Daniels IR, Smart NJ (2017) Systematic review and meta-analysis of the role of metronidazole in post-haemorrhoidectomy pain relief. Colorectal Dis 19(9):803–811. 10.1111/codi.13755 28589634 10.1111/codi.13755

[25] Re AD, Toh JWT, Iredell J, Ctercteko G (2020) Metronidazole in the management of post-open haemorrhoidectomy pain: systematic review. Ann Coloproctol 36(1):5–11. 10.3393/ac.2020.01.08 32146782 10.3393/ac.2020.01.08 PMC7069672

[26] Wanis KN, Emmerton-Coughlin HM, Coughlin S, Foley N, Vinden C (2017) Systemic metronidazole may not reduce posthemorrhoidectomy pain: a meta-analysis of randomized controlled trials. Dis Colon Rectum 60(4):446–455. 10.1097/DCR.0000000000000792 28267013 10.1097/DCR.0000000000000792

[27] Fishbein SRS, Mahmud B, Dantas G (2023) Antibiotic perturbations to the gut microbiome. Nat Rev Microbiol 21(12):772–788. 10.1038/s41579-023-00933-y 37491458 10.1038/s41579-023-00933-y PMC12087466

[28] Jakobsson HE, Jernberg C, Andersson AF, Sjölund-Karlsson M, Jansson JK, Engstrand L (2010) Short-term antibiotic treatment has differing long-term impacts on the human throat and gut microbiome. PLoS ONE 5(3):e9836. 10.1371/journal.pone.0009836 20352091 10.1371/journal.pone.0009836 PMC2844414

